# Comparision of Piceid and Resveratrol in Antioxidation and Antiproliferation Activities *In Vitro*

**DOI:** 10.1371/journal.pone.0054505

**Published:** 2013-01-16

**Authors:** Dan Su, Ying Cheng, Miao Liu, Daozhou Liu, Han Cui, Bangle Zhang, Siyuan Zhou, Tiehong Yang, Qibing Mei

**Affiliations:** 1 Department of Pharmaceutics, School of Pharmacy, Fourth Military Medical University, Xi’an, People’s Republic of China; 2 National Key Lab Of Gastrointestinal Pharmacology of Chinese Medicine, School of Pharmacy, Fourth Military Medical University, Xi’an, People’s Republic of China; National Institutes of Health, United States of America

## Abstract

**Background:**

The clinic therapeutic effect of resveratrol is limited due to its low oral bioavailability. Piceid, a precursor of resveratrol, is the most abundant form of resveratrol in nature. A number of studies have hypothesized that piceid may have the same bioactivities like those of resveratrol. The aim of this work is to compare piceid with resveratrol in antioxidation and antiproliferation activities *in vitro*.

**Methods:**

The antioxidative effects of resveratrol and piceid were evaluated by phenanthroline-Fe^2+^ method and H_2_O_2_-induced oxidative injury cell model. The antiproliferation effects were determined by MTT method in human liver tumor HepG2 cells, human breast cancer MDA-MB-231 cells and MCF-7 cells. The effects of resveratrol and piceid on the cell cycle and the apoptosis were evaluated by flow cytometry. Additionally, the uptake profiles of resveratrol and piceid in cancer cells were observed using fluorescence microscopy and clarified by LC-MS/MS.

**Conclusion:**

Piceid exhibited higher scavenging activity against hydroxyl radicals than resveratrol *in vitro*. Resveratrol showed a significant protective effect against H_2_O_2_-induced cell damage. What is more, resveratrol had biphasic effects on tumor cells. Resveratrol and piceid only showed significant cytotoxicity on tumor cells at high concentration (≥50 µmol/L), while low concentration of resveratrol (<30 µmol/L) increased the cell viability. The principal effect of resveratrol and piceid on the viability of tumor cells was caused by the cell cycle arrest, while the effect on apoptosis was relatively minor. The reason that piceid showed lower biological activity than resveratrol at the same concentration was probably because piceid was more difficult in being uptaken by cells.

## Introduction

Resveratrol (trans-3,5,4′-trihydroxystilbene) and piceid (resveratrol-3-O-β-mono- D-glucoside, the glycoside form of resveratrol) are the main active components of *Polygonum cuspidatum*, which is a traditional Chinese medicine. This medicine has been used for thousands of years in the treatment of neuropsychiatric disorders [1], [2]. *In vitro* researches indicate that resveratrol has chemoprevention effects against cardiovascular diseases, aging, and cancer [3], [4]. However, the absorption and metabolism of resveratrol have been extensively investigated by different research approaches. The oral bioavailability of resveratrol is extremely low due to rapid and extensive metabolism and the consequent formation of various metabolites such as resveratrol-glucuronides and resveratrol-sulfates [5], [6], [7]. Thus, *in vivo* experiments have yielded either positive or negative results. Some researchers conclude that resveratrol has no effects whereas others find it has a mild response [8], [9], [10], [11].

Piceid is a precursor of resveratrol. The average content of piceid was found ten times higher than that of resveratrol in the *Polygonum cuspidatum* and red wine [12]. What is more, piceid was the most abundant form of resveratrol in nature [13]. A number of studies have suggested that piceid, like resveratrol, may have the similar bioactivities such as anticarcinogenic effects [14], inhibition of platelet aggregation [15], [16], and antioxidation activity [17]. Recently, it is found that both piceid and resveratrol have antiinflammatory activity that can decrease IL-17 production in a concentration-dependent manner *in vitro* [18]. Up to now, it is still unclear whether piceid, like resveratrol, has the similar antioxidation and antitumor activities or possesses potential therapeutic advantage in clinic.

The aim of this work is to investigate the *in vitro* antioxidation and antiproliferation effects of resveratrol and piceid as well. The antioxidative effect of resveratrol and piceid was evaluated by phenanthroline-Fe^2+^ method and H_2_O_2_-induced oxidative injury HUVEC cell model. The effects of resveratrol and piceid on viability of tumor cells were determined by MTT method. The effects of resveratrol and piceid on the cell cycle and the apoptosis were evaluated by flow cytometry. Additionally, the uptake profiles of resveratrol and piceid in cancer cells were observed through fluorescence microscopy and clarified by liquid chromatography tandem mass spectrum (LC-MS/MS).

## Materials and Methods

### Chemicals

Dulbecco’s Modified Eagle’s medium (DMEM) was purchased from GIBCO Company. Fetal bovine serum was supplied by Hyclone Company. *Trans*-piceid and *trans*-resveratrol, with a purity grade higher than 98%, were supplied by the Xi’an Xinrui Biological Engineering Technology Company. MTT was purchased from Amersco Company. Phenanthroline, ethanol, ferrisulphas, vitamin C and hydrogen peroxide are all analytical grade. The aqueous solutions were prepared with quality milliQ water.

### Cell Culture

Human umbilical vein endothelial cells (HUVEC) [19], [20], human liver tumor HepG2 cell line [21], [22], human breast cancer MDA-MB-231 cell line and MCF-7 cell line [23], [24], [25] were obtained from Shanghai Institutes for Biological Sciences, Chinese Academy of Science. The cells were cultured in DMEM medium supplemented with 10% heat-inactivated fetal calf serum, 100 U/mL penicillin and 100 µg/mL streptomycin, in a humidified incubator with 5% CO_2_ at 37°C. Routine examination was done for mycoplasma contamination. Cells in logarithmic growth phase were used for further experiments.

### Hydroxyl Radical Scavenging Capacity Assay

The hydroxyl radical-scavenging activity of resveratrol and piceid was measured according to the method of Jin et al [26] with some modifications. In this system, hydroxyl radicals were generated by the Fenton reaction. Hydroxyl radicals oxidize Fe^2+^ into Fe^3+^. Only Fe^2+^ is able to combine with 1,10-phenanthroline to form a red compound (1,10-phenanthroline-Fe^2+^) with the maximum absorbance at 536 nm. The concentration of hydroxyl radical is reflected by the degree of decolourization of the reaction solution. Briefly, typical reactions were started by addition of 1 mL phenanthroline alcohol solution (0.75 mmol/L) to a tube containing 2 mL phosphate buffer (0.2 mmol/L), 1 mL deionized water, 1 mL ferrisulphas (0.75 mmol/L) and 1 mL H_2_O_2_ solution (0.01%). Reaction was carried out for 60 min at 37°C. After that, the absorbance of reaction mixture was measured at 536 nm (AP). The reaction mixture without H_2_O_2_ was used as the blank (AB). When the deionized water was replaced by different concentrations of resveratrol, piceid and vitamine C,the absorbance of reaction mixture was recorded (AS). The radical scavenging rate (d) was calculated according to the following formula: d = (AS-AP)/(AB-AP) [16].

### The Protection Effect on Hydrogen Peroxide Damaged HUVEC Cells and MDA-MB-231 Cells

It was reported that resveratrol could reduce the lipid peroxidation and the risk of cardiovascular disease. HUVEC cell is endothelial cells of human umbilical vein. MDA-MB-231 cell is a human breast cancer cell lines. In order to observe the antioxidative effect on normal cells and tumor cells, both of two cell lines were used to study anti lipid peroxidation activity of resveratrol and piceid. Briefly, HUVEC cells or MDA-MB-231 cells were seeded into 96-well plates (1×10^4^ cells/well) and were randomly divided into three groups: normal group, model group and drug treated group. Cells in the normal group were incubated under the normal growth conditions. The HUVEC cells in the model group were incubated with H_2_O_2_ (100 µmol/L) for 24 h. In the drug treated group, the cells were pre-incubated for 4 h with different final concentrations of resveratrol and piceid, followed by 24 h incubation with H_2_O_2_ (100 µmol/L). The wells were then washed three times with phosphate-buffered saline (PBS) and incubated again for 4 h with adding 180 µL RPMI 1640 and 20 µL of MTT solution (5 mg/mL). After removing the culture medium, 150 µL DMSO was added to dissolve the precipitate, and the absorbance at 570 nm of the resulting solutions was measured using a CODA Automated EIA Analyzer (Bio-Rad Laboratories, Hercules, CA, USA).

### *In vitro* Cytotoxicity Assay

The cytotoxicity of resveratrol and piceid on HepG2 cell, MDA-MB-231 cell and MCF-7 cell were assessed by MTT method. Cells were cultured in RPMI 1640 medium supplemented with 10% fetal bovine serum, 100 U/mL penicillin and 100 µg/mL streptomycin at 37°C under 5% CO_2_. The cells were seeded into 96-well plates (1×10^4^ cells/well) and incubated for 24 h. The medium then was replaced with fresh medium containing serially diluted resveratrol or piceid (final DMSO was 0.3%, v/v), and plates were incubated for 48 h or 72 h. The wells were then washed three times with PBS and incubated again for 4 h with adding 180 µL RPMI 1640 and 20 µL of MTT solution (5 mg/mL). After removing the culture medium, 150 µL of DMSO was added to dissolve the precipitate, and the absorbance at 570 nm of the resulting solutions was measured using a CODA Automated EIA Analyzer (Bio-Rad Laboratories, Hercules, CA, USA).

### Cell Cycle Analysis

HepG2 cells, MDA-MB-231 cells and MCF-7 cells grown in six-well plates were treated with varying concentrations of resveratrol or piceid for 24 h. At the end of treatment, cells were trypsinized, washed twice with cold PBS and centrifuged. The cell pellet was resuspended in 50 µL cold PBS and fixed in 2 mL of 70% ice-cold ethanol. Cells were centrifuged and treated with 0.1% Triton X-100 for 5 min. After incubation, cells were centrifuged and resuspended in 1 mL of PBS. Ribonuclease (100 µg/mL) was then added and the cells were incubated at 37°C for 30 min. After further centrifugation, cells were resuspended in 1 mL of PBS containing 50 µg/mL propidium iodide (PI, Sigma) and incubated for 30 min at 4°C. The cells were analyzed by flow cytometry (Becton Dickinson FACScan). This experiment was performed four times.

### Apoptosis Analysis

Three million cells were incubated in a 60-mm tissue culture dish containing resveratrol or piceid for 48 h. Cells were harvested by trypsinization and centrifugation, then analyzed in a Becton Dickinson FACScan (excitation at 488 nm) equipped with Cell Quest software after staining with annexin V-FITC and propidium iodide. Apoptotic cells stained with annexin V (early apoptosis) or with both annexin V and propidium iodide (late apoptosis), necrotic cells stained with propidium iodide, and living cells did not contain either stain.

### Fluorescence Microscopy Experiment

Because resveratrol and piceid themselves have green fluorescence, the uptake of resveratrol and piceid by HepG2 cells and MDA-MB-231 cells were investigated by using fluorescence microscopy. HepG2 cells and MDA-MB-231 cells were seeded into 24-well plates (1×10^5^ cells/mL), with each well containing a coverglass. After 24 h, 20 µmol/L resveratrol or piceid (dissolved in serum-free medium) was added, and the cells were incubated for 5 min or 15 min at 37°C, then the medium was removed and the cells were washed with ice-cold PBS three times. Cells were fixed with freshly prepared 2.5% paraformaldehyde in PBS for 10 min. The cells were washed with ice-cold PBS three times again. Intrinsic fluorescence of resveratrol or piceid was observed using the fluorescence microscope (Carl Zeiss Canada Inc.).

### HPLC-MS/MS Analysis

HepG2 cells and MDA-MB-231 cells were seeded in 6 well plates at a density of 1×10^5^ cells/mL. The culture medium was replaced with fresh medium 24 h before the uptake experiments. Resveratrol or piceid were added to culture medium. At end of the culture, the medium was removed and the cells were washed with ice-cold PBS three times. Then cells were collected and resuspended in 1 mL water. To determine the intracellular concentration of resveratrol or piceid, the cell lysate was obtained by subjecting the drug-containing cells to three freeze-thaw cycles in liquid nitrogen. Then the cell lysate was centrifugated at 12000 rpm and the supernatant was used for HPLC-MS/MS analysis. Protein concentrations in cell lysate were measured by using the Bradford method with bovine serum albumin as a standard.

### HPLC-MS/MS Condition

Resveratrol and piceid in cell lysate were analyzed as previously described [27]. A Quattro Premier liquid chromatography-mass/mass (LC-MS/MS) system (Waters Corp., Milford, MA) operating under Masslynx 4.1 software was used. Briefly, an XTerra C_18_ analytical column (150×2.1 mm, 5 µm, Waters Corp.) was used with the mobile phase gradient elution of acetonitrile/water. Eluant A was acetonitrile, and eluant B was water. The gradient was run from 30% A to 100% B over 6 min. An electrospray ionization-mass spectrometry (ESI^-^) source was used and was operated in negative ion mode in selected multiple reaction monitoring (MRM) mode. The source temperature was 110°C, and the desolvation temperature was 300°C. Nitrogen was used as the desolvation and cone gas with a flow rate of 650 and 50 L/h, respectively. Argon was used as the collision gas at a rate of 0.18 mL/min. The auto-sampler was maintained at 25°C and the injection volume was 20 µL. The mass spectrum conditions of resveratrol and piceid was shown in table 1. Under the optimized chromatographic condition, the analytical method was linear in the range of 0.4 to 300 ng/mL with an average correlation coefficient of 0.9986.

**Table 1:** Mass spectral conditions for triple quadrupole mass spectrometer.

compound	Parent(m/z)	Daughter(m/z)	Capillary voltage(kV)	Cone Voltage(V)	Collision energy(eV)
resveratrol	229.0	134.8	3.0	37	17
piceid	390.8	228.8	3.0	16	8

### Statistical Analysis

All data were processed and analyzed by Sigma-Plot 8.0 software. The statistical significances were evaluated by t-test of the software and p<0.05 was considered significant.

## Results

### Hydroxyl Radical Scavenging Capacity in Phenanthroline-Fe^2+^ System

**Figure 1:**
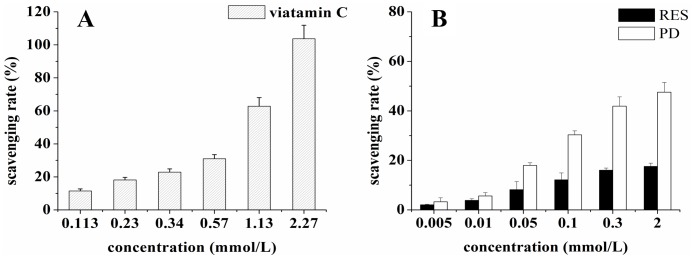
The hydroxyl radical scavenging capacity of vitamine C (panel A), resveratrol (RES) and piceid (PD) (panel B) in phenanthroline-Fe^2+^ system.

Hydroxyl radicals generated in the Fe^2+^/H_2_O_2_ system were determined by a spectrophotometric method. The hydroxyl radical scavenging capacity of resveratrol and piceid are showed in figure 1. It was indicated that both resveratrol and piceid exhibited the capacity of scavenging hydroxyl radicals. Piceid showed higher scavenging activity against hydroxyl radicals than resveratrol did. When the scavenging rate was 12%, the concentration of resveratrol was 0.1 mmol/L, the concentration of piceid was lower than 0.05 mmol/L, while the concentration of vitamine C was more than 0.113 mmol/L. This indicated that resveratrol and piceid had higher hydroxyl radical scavenging capacity than vitamine C at low concentration. When the concentration was higher than 0.1 mmol/L, the dissolution of resveratrol and piceid got saturated, which resulted in the saturation of their hydroxyl radicals scavenging capacity at high concentration.

### The Protection Effect on HUVEC Cells and MDA-MB-231 Cells Injured by Hydrogen Peroxide

**Figure 2:**
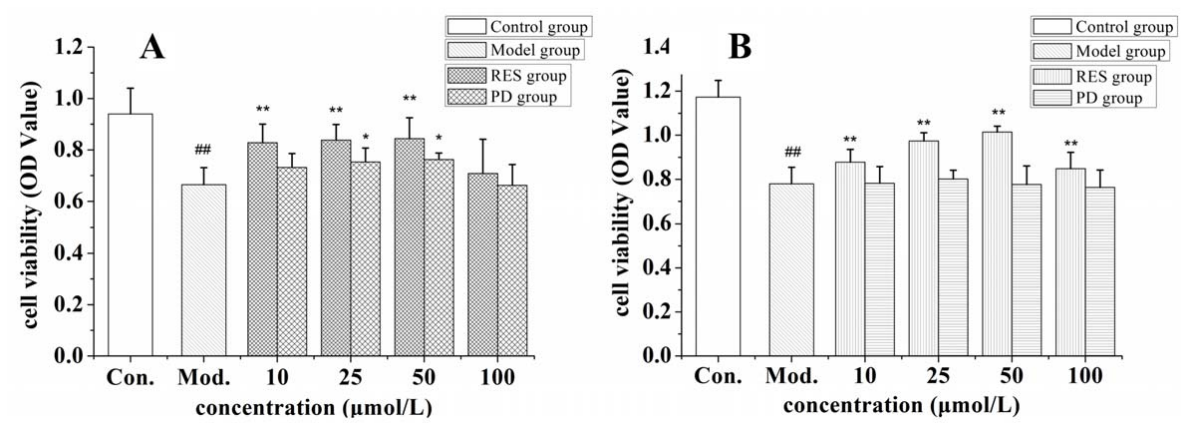
The protective effect of resveratrol (RES) and piceid (PD) on oxidation damage of HUVEC cells (panel A) and MDA-MB-231 cells (panel B) injured by 100 µmol/L hydrogen peroxide. Compared with control group (Con.), ^##^p<0.01; compared with model group (Mod.), *p<0.05, **p<0.01.

Oxidative stress induced by 100 µmol/L H_2_O_2_ was used to determine the anti lipid peroxidation activities of resveratrol and piceid in HUVEC cells and MDA-MB-231 cells. The results are showed in figure 2. Cell viability was respectively decreased 30% and 33% by incubation 100 µmol/L H_2_O_2_ with HUVEC cells and MDA-MB-231 cells for 24 h. Resveratrol significantly improved cell viability when the concentration ranged from 10 to 50 µmol/L in two cell lines, while its protection effect decreased at concentration of 100 µmol/L. Piceid showed the same effect like resveratrol on the viability of HUVEC cells. Piceid did not increase the viability of MDA-MB-231 cells when concentration ranged from 10 to 100 µmol/L.

### Cytotoxicity on Tumor Cells

**Figure 3:**
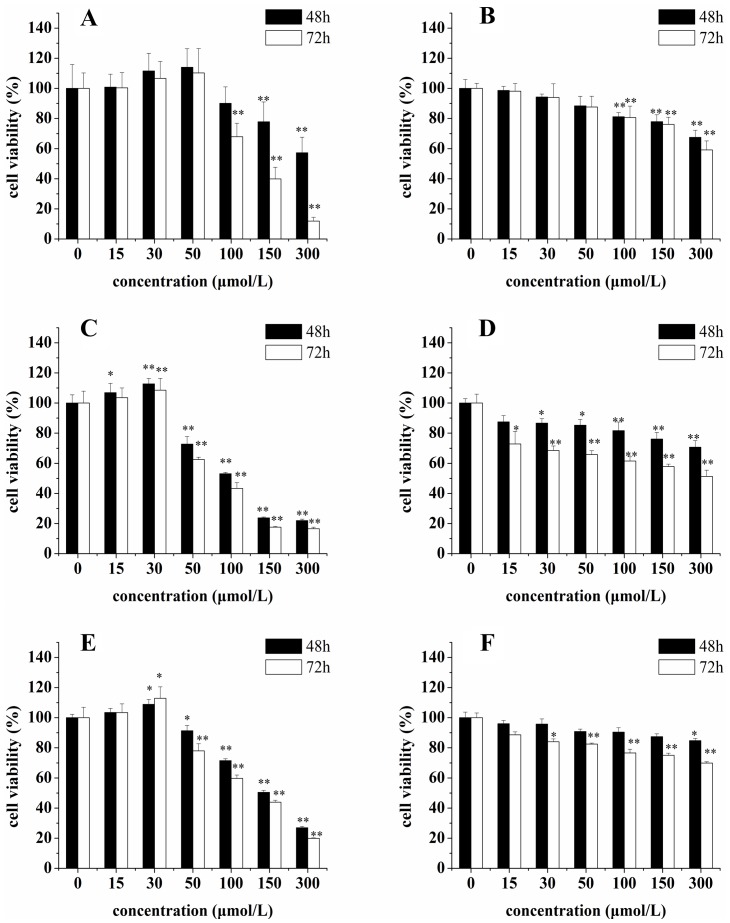
Effect of resveratrol (RES) and piceid (PD) on the cell viability of HepG2 cells (panel A-RES, panel B-PD), MDA-MB-231 cells (panel C-RES, panel D-PD) and MCF-7 cells (panel E-RES, panel F-PD). Compared with control group, *p<0.05, **p<0.01.

The cytotoxicity of resveratrol and piceid on three cancer cell lines was examined by MTT method, the results of which are showed in figure 3. When the concentrations were lower than 30 µmol/L, neither resveratrol or piceid showed significant cytotoxicity on HepG2 cells, MCF-7 cells and MDA-MB-231 cells. Actually, resveratrol even increased the viability of HepG2 cells, MCF-7 cells and MDA-MB-231 cells at lower concentrations. Unlike resveratrol, piceid did not show the same effect. However, when concentrations were higher than 50 µmol/L, resveratrol and piceid showed significant cytotoxicity on HepG2 cells, MCF-7 cells and MDA-MB-231 cells in concentration- and time-dependent manner. Resveratrol showed higher inhibition capacity in cell viability than piceid did. Moreover, resveratrol and piceid showed greater inhibition capacity in cell viability in MDA-MB-231 cells than that in HepG2 cells or MCF-7 cells.

### Cell Apoptosis Assessed by Flow Cytometry

**Figure 4:**
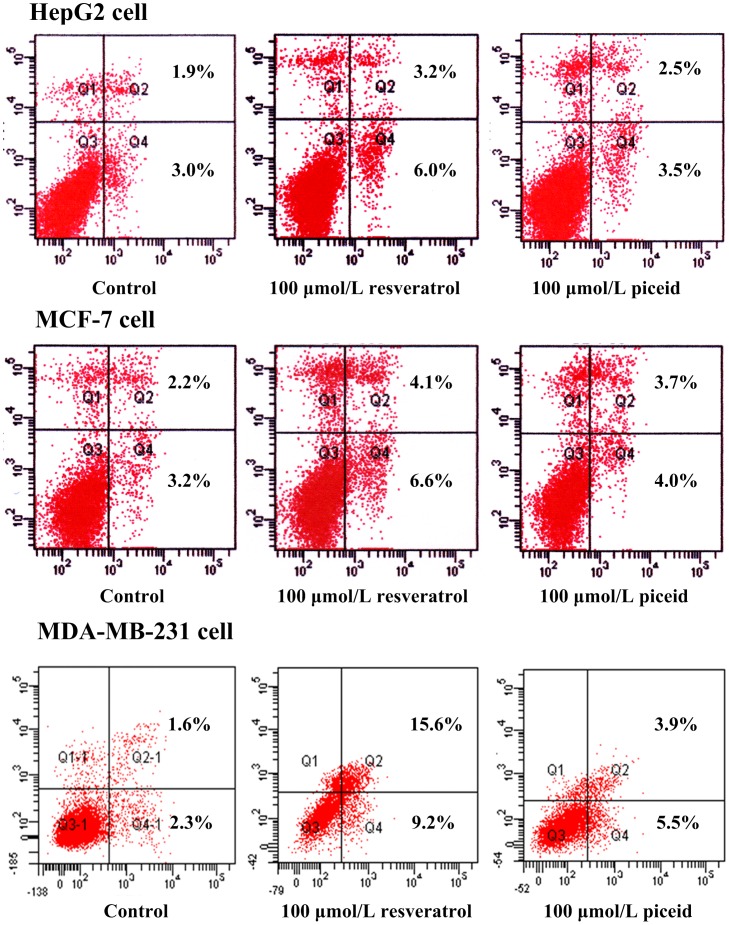
Tumor cells apoptosis induced by resveratrol and piceid. Cultures of HepG2 cells, MCF-7 cells and MDA-MB-231 cells were treated with 100 µmol/L resveratrol and piceid for 48 h. Cells were harvested by trypsinization and centrifugation, then analyzed in a Becton Dickinson FACScan (excitation at 488 nm) equipped with Cell Quest software after staining with annexin V-FITC and propidium iodide. Results shown are of an experiment representative of apoptosis.

For cell apoptosis analysis, HepG2 cells, MCF-7 cells and MDA-MB-231 cells were incubated with different concentrations of resveratrol or piceid (10, 50, 100 and 300 µmol/L) for 48 h. The percentage of apoptosis at the early and late phases was determined by flow cytometry. Representative pictures are showed in figure 4. The results are respectively showed in table 2, table 3, and table 4. Significant cells apoptosis was not observed in HepG2 cells and MCF-7 cells at concentrations below 100 µmol/L. While at 100 µmol/L, resveratrol induced the significant apoptosis in MDA-MB-231 cells. At 300 µmol/L, resveratrol induced the significant apoptosis in all three tumor cell lines. Piceid only induced the significant apoptosis in MDA-MB-231 cells at the concentration of 100 µmol/L and 300 µmol/L. More apoptosis was observed in MDA-MB-231 cells than that in HepG2 cells and MCF-7 cells when they were treated with resveratrol or piceid.

**Table 2:** The HepG2 cells apoptosis induced by resveratrol and piceid.

	Control	Resveratrol (µmol/L)				Piceid (µmol/L)			
		10	50	100	300	10	50	100	300
Late apoptosis rate (%)	1.7±0.5	2.0±0.6	2.1±0.5	3.2±1.1	4.4±1.5*	1.8±0.4	2.3±0.7	2.5±0.8	3.0±0.9
Early apoptosis rate (%)	4.0±1.5	5.0±1.6	5.4±1.2	9.0±3.0	13.7±2.6*	3.7±0.8	3.9±0.7	4.5±1.5	5.6±1.4
Total apoptosis rate (%)	5.7±1.9	7.0±1.9	7.5±2.2	12.2±3.2	18.1±4.1**	5.5±1.1	6.1±1.8	7.0±2.3	8.7±2.1

* P<0.05 *vs* control,

** P<0.01 *vs* control, n = 3.

**Table 3:** The MCF-7 cells apoptosis induced by resveratrol and piceid.

	Control	Resveratrol (µmol/L)				Piceid (µmol/L)			
		10	50	100	300	10	50	100	300
Late apoptosis rate (%)	2.7±0.7	2.6±0.8	3.2±0.4	5.2±1.2	6.1±1.9*	2.1±0.5	3.4±1.1	3.5±1.3	3.9±1.3
Early apoptosis rate (%)	3.2±1.2	3.0±1.1	4.8±1.5	8.5±2.0	14.2±3.0**	3.0±1.2	4.2±1.7	4.6±1.7	6.0±1.8
Total apoptosis rate (%)	5.9±1.7	5.6±1.6	7.8±2.3	13.7±3.2	20.3±4.7**	5.1±1.8	7.6±2.6	8.1±3.0	9.9±2.9

* P<0.05 *vs* control,

** P<0.01 *vs* control, n = 3.

**Table 4:** The MDA-MB-231 cells apoptosis induced by resveratrol and piceid.

	Control	Resveratrol (µmol/L)				Piceid (µmol/L)							
		10	50	100	300	10		50		100		300	
Late apoptosis rate (%)	2.9±0.9	2.2±0.6	4.1±1.2	17.7±2.4*	20.6±3.9**	2.8±0.8	3.9±0.7		5.7±2.0			9.4±2.5*	
Early apoptosis rate (%)	1.2±0.6	2.1±0.5	5.8±1.5*	13.0±2.5*	23.2±4.1**	1.1±0.4		4.1±1.3	6.7±1.4*		12.8±2.9*		
Total apoptosis rate (%)	4.1±1.7	4.3±1.8	9.9±2.4**	30.7±4.9**	43.8±8.5**	3.9±1.4	7.9±2.2		12.4±2.4*			22.2±3.9**	

* P<0.05 *vs* control,

** P<0.01 *vs* control, n = 3.

### Effect of Resveratrol and Piceid on Cell Cycle

**Figure 5:**
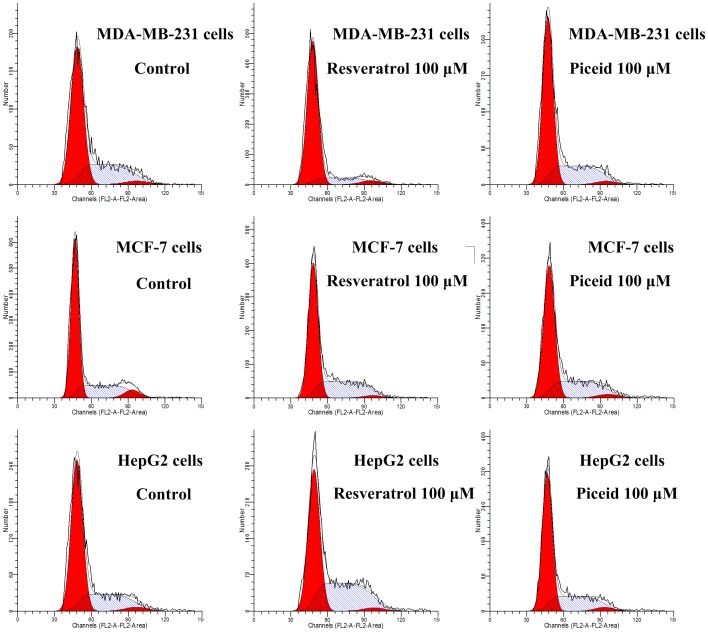
Effects of resveratrol (RES) and piceid (PD) on cell cycle of MDA-MB-231 cells, MCF-7 cells and HepG2 cells. Cultures of MDA-MB-231 cells were treated with varying concentrations of resveratrol and piceid for 24 h. At the end of treatment, cells were trypsinized, incubated with RNase, stained with propidium iodide (PI), and analyzed by flow cytometry. Results shown are of an experiment representative of cell cycle.

To further probe into the nature of growth inhibition by resveratrol and piceid, cell cycle was analyzed by flow cytometry. After the cells were treated with resveratrol or piceid for 24 h, their distribution in different phases of the cell cycle is illustrated in table 5, table 6, and table 7. Representative pictures are showed in figure 5. The results showed that for HepG2 cells and MCF-7 cells, the percentage of cells in the G1 phase was reduced by incubating with resveratrol or piceid. This reduction was accompanied by an increase in the proportion of cells in the S phase in concentration-dependent manner. For MDA-MB-231 cells, the percentage of cells in the G1 phase was increased in concentration-dependent manner. This increase was accompanied by a reduction in the proportion of cells in the S phase. Compared with piceid, resveratrol showed greater effects on cell cycle.

**Table 5:** Effect of resveratrol and piceid on MCF-7 cells cycle.

	Control	Resveratrol (µmol/L)				Piceid (µmol/L)			
		10	50	100	300	10	50	100	300
G1	64.8±8.3	60.6±8.6	55.2±9.0	53.6±5.5	52.3±9.1	64.6±7.1	63.2±9.7	62.0±6.5	61.6±9.5
S	28.8±3.4	37.0±5.5	41.4±4.7*	42.5±4.1*	43.2±5.9*	31.8±6.5	33.4±6.7	35.1±4.3	36.2±7.8
G2	6.4±2.9	2.4±1.9	3.4±1.9	3.9±1.7	4.5±2.1	3.6±1.1	3.3±1.8	2.9±1.5	2.2±1.9

* P<0.05 *vs* control, n = 4.

**Table 6:** Effect of resveratrol and piceid on HepG2 cells cycle.

	Control	Resveratrol (µmol/L)				Piceid (µmol/L)			
		10	50	100	300	10	50	100	300
G1	63.9±9.9	58.5±7.8	53.5±7.1	49.3±4.5	47.1±8.7	64.3±8.9	60.4±8.4	58. 9±5.2	55. 9±8.7
S	29.7±3.2	33.2±5.9	41.2±3.1*	45.8±3.4*	48.6±5.3**	31.4±5.5	35.8±6.3	36.8±3.4	39.7±7.3
G2	6.4±1.7	6.3±1.9	5.3±2.3	4.9±1.2	4.3±1.5	5.3±1.8	4.8±2.3	4.3±2.1	4.4±2.9

* P<0.05 *vs* control,

** P<0.01 *vs* control, n = 4.

**Table 7:** Effect of resveratrol and piceid on MDA-MB-231 cells cycle.

	Control	Resveratrol (µmol/L)				Piceid (µmol/L)			
		10	50	100	300	10	50	100	300
G1	66.2±5.1	71.4±8.6	76.2±5.2*	82.4±4.8*	84.0±6.7**	65.3±7.6	71.5±8.9	77.1±5.3	78.9±6.2*
S	30.4±7.4	27.0±5.4	20.5±4.2	14.1±1.6	12.6±3.7	32.0±5.5	25.5±4.7	19.6±2.1	17.9±4.5
G2	3.4±1.8	1.6±1.2	3.3±2.2	3.5±2.2	3.4±2.1	2.7±1.8	3.0±1.9	3.3±1.9	3.2±2.1

* P<0.05 *vs* control,

** P<0.01 *vs* control, n = 4.

### Fluorescence Microscopy Analysis of Intracellular Uptake

**Figure 6:**
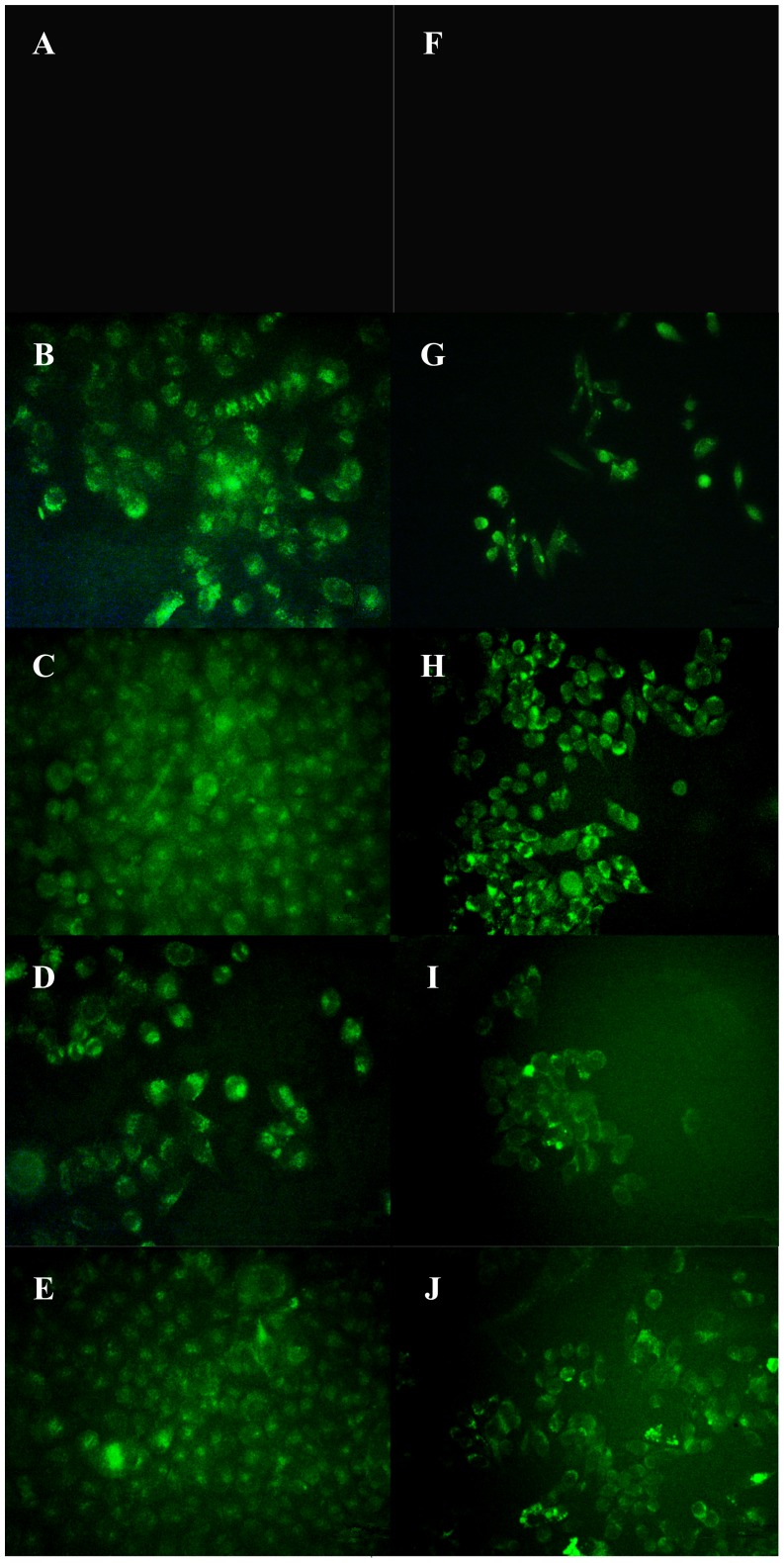
Observation of resveratrol (RES) and piceid (PD) uptake in HepG2 cells (A, B, C, D, E) and MDA-MB-231 cells (F, G, H, I, J) by fluorescence microscopy. Panel A and panel F-negative control; Panel B and panel G-incubating with resveratrol for 5 min; Panel C and panel H -incubating with resveratrol for 15 min; Panel D and panel I -incubating with piceid for 5 min; Panel E and panel J -incubating with piceid for 15 min. The concentration of resveratrol or piceid is 20 µmol/L.

Resveratrol and piceid intrinsic green fluorescence allowed a direct visualization of their intracellular uptake. A green fluorescence was observed in drug treated cells, markedly higher than basal fluorescence in untreated cells. The cellular uptake of resveratrol and piceid in HepG2 cells and MDA-MB-231 cells is showed in figure 6. The result showed that fluorescence was distributed throughout the whole cell. With the increase of incubation time, the intensity of ﬂuorescence observed in HepG2 cells and MDA-MB-231 cells increased significantly, indicating that more resveratrol and piceid were uptaken by cells as incubation time prolonged.

### The Quantitative Uptake of Resveratrol and Piceid in HepG2 and MDA- MB-231cells

**Figure 7:**
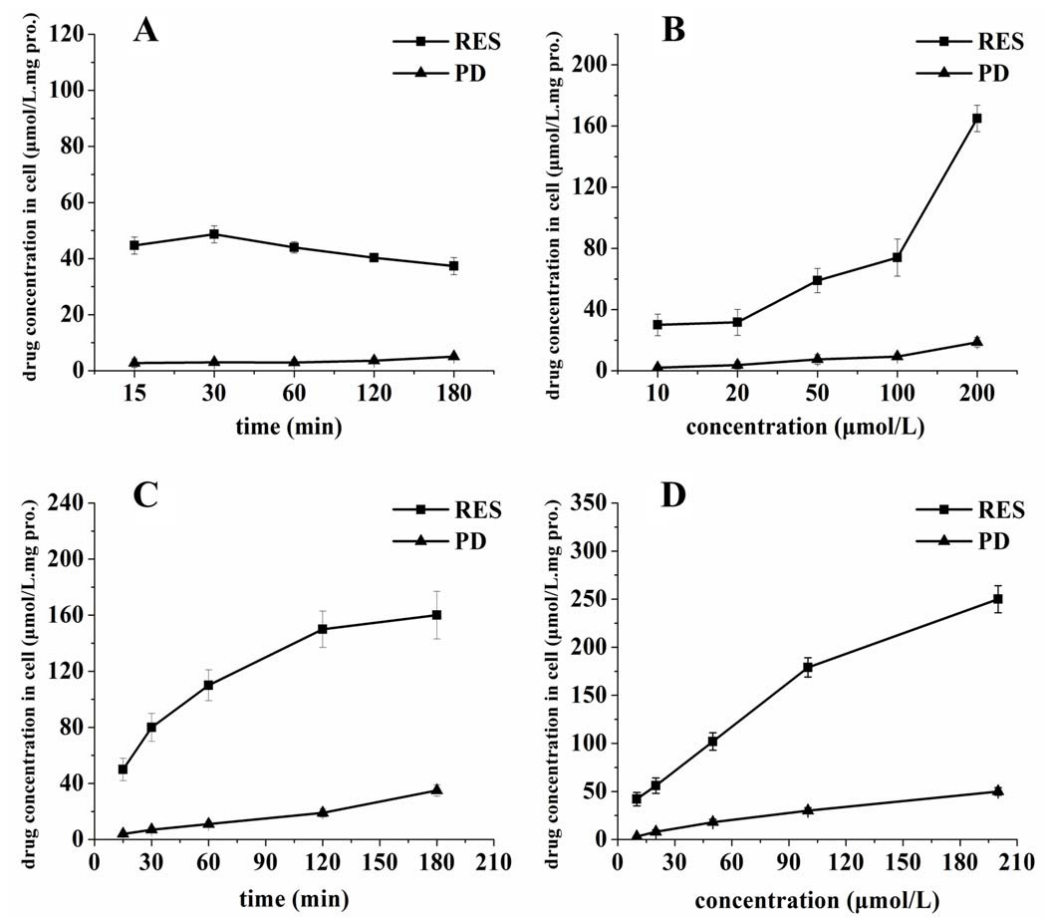
The quantitative uptake curve of resveratrol (RES) and piceid (PD) after incubated with HepG2 cells (A and B) and MDA-MB-231 cells (C and D). Panel A and panel C show the time course uptake of RES and PD at the concentration of 15 µmol/L, Panel B and panel D show concentration-dependent uptake of RES and PD in 30 min.

To quantitatively examine the time-course uptake characteristics of resveratrol and piceid, HepG2 cells and MDA-MB-231 cells were incubated with 15 µmol/L resveratrol or piceid at different duration time. The dose dependent uptake was examined by incubating the cells with various concentrations of resveratrol or piceid. The results are showed in figure 7.

The intracellular accumulation of resveratrol in HepG2 cells was increased rapidly during the first 30 min and reached 48.6 µmol/L mg.protein. Thereafter, the intracellular concentration of resveratrol in HepG2 cells was decreased gradually, which indicated the rate of resveratrol cumulated in the cells was slower than that of resveratrol eliminated from the cells. The time-course characteristic of piceid uptake was different from resveratrol in HepG2 cells. With the prolonging of incubation time, intracellular piceid level increased slowly. In MDA-MB-231 cells, the intracellular level of resveratrol was saturated at 180 min, the uptake of piceid did not reach saturation at 180 min. The amount of intracellular resveratrol in HepG2 cells and MDA-MB-231 cells was much higher than that of piceid in the same incubation time.

When the concentration of resveratrol and piceid increased from 10 µmol/L to 200 µmol/L, the intracellular level of resveratrol or piceid increased continuously. The amount of intracellular resveratrol was much higher than that of piceid when HepG2 cells and MDA-MB-231 cells were incubated with the same concentration of resveratrol or piceid. This showed that resveratrol was easier to be uptaken by cells than piceid. Compared with HepG2 cells, more resveratrol and piceid were accumulated in MDA-MB-231 cells.

## Discussion

Since 1997 when resveratrol was showed to have cancer chemopreventive activity in three major stages of carcinogenesis, many studies have focused on antioxidative, anticarcinogenic and antiinflammatory effects of resveratrol. Although piceid is the glycoside form of resveratrol, its antiproliferation and antioxidation activities are rarely reported.

The hydroxyl radical can damage virtually all types of macromolecules: carbohydrates, nucleic acids, lipids and amino acids. The hydroxyl radical is related to chronic health problems like cancer, arthrosclerosis and ageing [28]. Mechanisms for scavenging hydroxyl radical for the protection of cellular structures includes endogenous antioxidants such as melatonin and glutathione, and dietary antioxidants. The present study showed that both resveratrol and piceid were antioxidants and their hydroxyl radical scavenging capacity was greater than vitamine C at lower concentrations. Compared with resveratrol, piceid showed stronger hydroxyl radical scavenging capacity in phenanthroline-Fe^2+^ system.

Vascular endothelial cell injury is not only the initiating factor of atherosclerosis, but also plays an important role in the occurrence and development of coronary heart disease and high blood pressure. In this study, in order to evaluate the antioxidative effect on normal cells and tumor cells, hydrogen peroxide was incubated with HUVEC cells and MDA-MB-231 cells. The aim was to overproduce reactive oxygen species (ROS) and induce cells into oxidative stress condition. The results indicated that resveratrol significantly attenuated the oxidative damage and improved cell viability in both HUVEC cells and MDA-MB-231 cells when concentration ranged from 10 to 50 µmol/L. However, its protective effect decreased at the concentration of 100 µmol/L. Like resveratrol, piceid had the same effect on HUVEC cells. Piceid showed no significant protective effect on MDA-MB-231 cells when concentration ranged from 10 to 100 µmol/L. The antioxidative effect of resveratrol was related with the increase of superoxide dismutase, glutathione peroxidase, catalase activities, and directly scavenging the ROS in cells [29], [30].

The antioxidation activity of resveratrol can be traced back to its cancer chemoprevention since free-radical-mediated oxidative damage of DNA is involved with the development of cancer cells [31], [32], [33], [34]. In fact, low concentration of antioxidants is, probably, sufficient to correct low increases in intracellular reactive oxygen species (ROS), and in the long term, to delay the occurrence of the pre-malignant event in cancer progression and in the development of atherosclerotic plaques. On the other hand, more and more experimental evidence suggests that natural antioxidants play a chemopreventive role independent on their ability to scavenge reactive oxygen species [35], [36]. Furthermore, most free-radical scavengers can act both as antioxidants and pro-oxidants depending on the conditions [37]. Gallic acid and epigallocatechin gallate (EGCG) significantly increase the production of ROS. A study showed that EGCG neither directly scavenges H_2_O_2_ nor mediates other antioxidant activities, but increases H_2_O_2_-induced oxidative stress and DNA damage [38], [39]. There is evidence that some of the effects of catechins may be related to the induction of oxidative stress. Such pro-oxidant effects appear to be responsible for the induction of apoptosis in tumor cells. These pro-oxidant effects may also induce endogenous antioxidation systems in normal tissues that offer protection against carcinogenic insult [40].

A biphasic effect of resveratrol on tumor cells viability was observed in this research. At lower concentrations (≤30 µmol/L), the cell viability was increased by resveratrol in HepG2 cells, MCF-7 cells and MDA-MB-231 cells. However, at higher concentrations (≥50 µmol/L), the cytotoxicity of resveratrol and piceid increased in a dose-dependent manner. Resveratrol showed a stronger cytotoxicity than piceid. When cells were treated with resveratrol at the concentration of 50 µmol/L for 48 h, an average of 25%, 5% and 0% decrease in cell viability was observed respectively in MDA-MB-231 cells, MCF-7 cells and HepG2 cells, indicating that the cytotoxicity of resveratrol and piceid to MDA-MB-231 cells was greater than that to MCF-7 cells and HepG2 cells. The present data suggested that resveratrol and piceid had protective effect against oxidative stress damage at lower concentrations. On the other hand, resveratrol and piceid showed obvious cytotoxicity only at high concentration, indicating the effects of resveratrol are dose-dependent.

Stocco B et al investigated the antioxidative role of resveratrol on bladder carcinoma cell line ECV304. The results indicated that resveratrol at the concentration of 2.5 µmol/L protected the ECV304 cells from the damage induced by oxidative stress. High concentration (>20 µmol/L) of resveratrol induced cell death and increased cell damage from oxidative stress [41]. Beside, Bai Y et al investigated the chemopreventive potential of resveratrol against T24 cells [42]. It was showed that treatment of T24 cells with high concentration of resveratrol (>25 µmol/L) resulted in a significant decrease in cell viability by inducing apoptosis and cell cycle arrest. On the other hand, at low concentrations (<20 µmol/L), resveratrol was able to provide protection against various diseases, such as myocardial ischemic reperfusion injury, atherosclerosis, and ventricular arrhythmias [43]. In prostate cancer cells, the effect of resveratrol on DNA synthesis was dependent on the concentration and the duration of treatment. Resveratrol caused a 2- to 3-fold increase in DNA synthesis at the concentration of 5–10 µmol/L, but it inhibited DNA synthesis when its concentration was higher than 15 µmol/L [44]. Another study reported that low concentration (0.1–1.0 µg/mL) of resveratrol enhanced cell proliferation, higher concentration (10.0–100.0 µg/mL) induced apoptosis and decrease mitotic activity in colon cancer cells and endothelial cells [45]. This biphasic effect of resveratrol also was demonstrated on normal cells [46]. To sum up, our results clearly corroborated with other previous reports that diverse activities of resveratrol were decided by its concentration. Further studies are required to better characterize dual effects of resveratrol on cancer cells.

In order to get further information about the inhibition of resveratrol and piceid on tumor cells, flow cytometry was used to analyze the effect of piceid and resveratrol on cell apoptosis and cell cycle in HepG2 cells, MCF-7 cells and MDA-MB-231 cells. The results indicated that resveratrol and piceid reduced viability of tumor cells through cell cycle arrest and apoptosis induction. We found that piceid and resveratrol did not induce the apoptosis at the concentration of 50 µmol/L in HepG2 cells and MCF-7 cells. Meanwhile, resveratrol significantly induced apoptosis in MDA-MB-231 cells at the concentration of 50 µmol/L, indicating MDA-MB-231 cells was more sensitive to resveratrol and piceid than HepG2 cells and MCF-7 cells. Resveratrol obviously induced tumor cell apoptosis at high concentration (300 µmol/L) in all three kinds of tumor cells used in this research. Compared with piceid, resveratrol showed stronger apoptosis induction ability. Furthermore, our results showed that resveratrol induced the cell cycle arrest in the S phase in HepG2 cells and MCF-7 cells at concentration of 50 µmol/L. On the other hand, resveratrol induced the cell cycle arrest in the G1 phase in MDA-MB-231 cells at concentration of 50 µmol/L. Our results suggested that the principal effect of resveratrol and piceid on the proliferation inhibition of tumor cells was caused by the cell cycle arrest, while the effect on apoptosis was relatively minor.

Joe et al. found that higher dose of resveratrol (300 µmol/L) induced apoptosome formation and resulted in heightened cellular death [47]. This is contrasted with the cytotoxic effect of resveratrol at lower dose, which was unaffected by caspase inhibition and over-expression of antiapoptotic proteins [48]. It was found that the cytotoxic effects of resveratrol were seemingly equivalent in both normal and malignant cells at higher doses, whereas the cell death was specific for cancer cells at lower doses. As a result, there would be greater therapeutic potential in understanding and exploiting the mechanisms utilized between 50 and 100 µmol/L of resveratrol [8].

Goldberg orally administered resveratrol (25 mg/70 kg) to healthy volunteers. It was found that free resveratrol level in plasm was less than 40 nmol/L, and free resveratrol accounted for only a small fraction of the total dose in plasma (1.7–1.9%) [49]. A recent phase I dose escalation study evaluated the safety and pharmacokinetics of resveratrol administered as a single dose (0.5, 1.0, 2.5, or 5.0 g) in healthy volunteers. At the highest dose, the average peak plasma concentration (Cmax) of resveratrol was 539±384 ng/mL, which equated to 2.4 µmol/L [50], [51]. This indicated that circulating concentrations generated through consumption of dietary sources would be inadequate to elicit biological effects because the biological activity of resveratrol was observed between 10 and 100 µmol/L *in vitro* [52], [53]. We conclude that the widespread difficulty in translating *in vitro* results to *in vivo* models can be largely attributed to the inability of achieving and maintaining elevated serum concentrations of resveratrol.

In order to explain why resveratrol showed stronger biological activity than piceid, we investigated the uptake of piceid and resveratrol in the different tumor cells. The fluorescence microscopy images indicated that fluorescence distributed among the whole cell. We discovered that more resveratrol and piceid were uptaken by HepG2 and MDA-MB-231 cells as time prolonged. Our discovery was consistent with what Lançon A et al observed about the uptake of resveratrol (30 µmol/L) in HepG2 cells by fluorescence microscopy [21].

The LC-MS/MS was used to quantitatively determine the intracellular accumulation of piceid and resveratrol. The amount of the resveratrol in HepG2 cells reached saturation in 30 min whereas the amount of intracellular piceid still increased at 180 min. The uptake of piceid and resveratrol by HepG2 cells and MDA-MB-231 cells showed a time- and concentration-dependent manner, and the amount of resveratrol in HepG2 cells and MDA-MB-231 cells increased more quickly than piceid. This indicated piceid was harder to be uptaken by the cells than resveratrol. It may help to explain why piceid had lower biological activity than resveratrol. Compared with HepG2 cells, more resveratrol and piceid were uptaken by MDA-MB-231 cells. This maybe one of the reasons why MDA-MB-231 cells were more sensitive to resveratrol and piceid than HepG2 cells in terms of cell viability, cell cycle and apoptosis. Lançon A et al investigated into the uptake of resveratrol in HepG2 cells by liquid scintillation analyzer. The results indicated that the uptake of resveratrol by HepG2 cells showed a time- and concentration-dependent manner, and both passive diffusion and carrier-mediated processes were involved in resveratrol uptake by HepG2 cells [21], [22]. Various studies showed that resveratrol efflux involved these transporters, such as multidrug resistance-associated protein (MRP) and the breast cancer resistance protein (BCRP or ABCG2), playing an important role in the export of conjugated molecules (glucuronides or sulphates) [54], [55], [56]. In particular, lipid rafts endocytosis was a determinant process for the biological activities of resveratrol. This uptake pathway was associated with the involvement of integrin αvβ3 MAPK activation and p53-dependent apoptosis in a variety of cancer cells [57].

We conclude from the study that piceid has stronger radical scavenging capacity than resveratrol *in vitro*, but has no protective effect on hydrogen peroxide damaged MDA-MB-231 cells. Resveratrol has biphasic effects on tumor cells. Their underlying mechanisms should be further elucidated to help identify the optimum doses to be used in the clinical trials and evaluate the efficacy of resveratrol. Piceid has not biphasic effects on tumor cells. Resveratrol and piceid only show significant cytotoxicity on tumor cells at high concentration. This indicates that it is not easy to develop resveratrol and piceid as antitumor drug since it is difficult to achieve and maintain high serum concentrations of resveratrol and piceid *in vivo* [27], [57]. The principal effect of resveratrol and piceid on the proliferation inhibition of tumor cells was cell cycle arrest, with the effect on apoptosis being relatively minor. Piceid shows lower biological activity than resveratrol at the same concentration due to the fact that the uptake of the piceid by cells is harder than that of resveratrol.

In this study, the antioxidation and antiproliferation activities of resveratrol and picied were investigated *in vitro* at the concentration ranging from 10 to 300 µmol/L. The effects of resveratrol were probably not specific at high concentration. What is more, the effects of resveratrol are limited *in vivo* because it is difficult to achieve the concentration of 300 µmol/L in plasma after oral administration of resveratrol or piceid. The antioxidation and antitumor activities of resveratrol and picied should be further elucidated in animal model.
